# Regulation of Fn14 Receptor and NF-κB Underlies Inflammation in Meniere’s Disease

**DOI:** 10.3389/fimmu.2017.01739

**Published:** 2017-12-13

**Authors:** Lidia Frejo, Teresa Requena, Satoshi Okawa, Alvaro Gallego-Martinez, Manuel Martinez-Bueno, Ismael Aran, Angel Batuecas-Caletrio, Jesus Benitez-Rosario, Juan M. Espinosa-Sanchez, Jesus José Fraile-Rodrigo, Ana María García-Arumi, Rocío González-Aguado, Pedro Marques, Eduardo Martin-Sanz, Nicolas Perez-Fernandez, Paz Pérez-Vázquez, Herminio Perez-Garrigues, Sofía Santos-Perez, Andres Soto-Varela, Maria C. Tapia, Gabriel Trinidad-Ruiz, Antonio del Sol, Marta E. Alarcon Riquelme, Jose A. Lopez-Escamez

**Affiliations:** ^1^Otology and Neurotology Group CTS495, Department of Genomic Medicine – Centre for Genomics and Oncological Research – Pfizer/Universidad de Granada/Junta de Andalucía (GENYO), Granada, Spain; ^2^Computational Biology Group, Luxembourg Centre for Systems Biomedicine (LCSB), Universite du Luxembourg, Belval, Luxembourg; ^3^Group of Genetics of Complex Diseases, Department of Genomic Medicine – Centre for Genomics and Oncological Research – Pfizer/Universidad de Granada/Junta de Andalucía (GENYO), Granada, Spain; ^4^Department of Otolaryngology, Complexo Hospitalario de Pontevedra, Pontevedra, Spain; ^5^Department of Otolaryngology, Hospital Universitario Salamanca, IBSAL, Salamanca, Spain; ^6^Department of Otolaryngology, Hospital Universitario de Gran Canaria Dr Negrin, Las Palmas de Gran Canaria, Las Palmas, Spain; ^7^Department of Otolaryngology, Instituto de Investigación Biosanitaria ibs.GRANADA, Hospital Universitario Virgen de las Nieves, Granada, Spain; ^8^Department of Otolaryngology, Hospital Miguel Servet, Zaragoza, Spain; ^9^Department of Otorhinolaryngology, Hospital Universitario Vall d’Hebron, Barcelona, Spain; ^10^Department of Otorhinolaryngology, Hospital Universitario Marqués de Valdecilla, Santander, Cantabria, Spain; ^11^Department of Otorhinolaryngology, Centro Hospitalar de S.João, EPE, University of Porto Medical School, Porto, Portugal; ^12^Department of Otolaryngology, Hospital Universitario de Getafe, Getafe, Madrid, Spain; ^13^Department of Otolaryngology, Clínica Universidad de Navarra, Pamplona, Spain; ^14^Department of Otorhinolaryngology, Hospital Universitario de Cabueñes, Gijón, Asturias, Spain; ^15^Department of Otorhinolaryngology, Hospital La Fe, Valencia, Spain; ^16^Division of Otoneurology, Department of Otorhinolaryngology, Complexo Hospitalario Universitario, Santiago de Compostela, Spain; ^17^Department of Otorhinolaryngology, Instituto Antolí Candela, Madrid, Spain; ^18^Division of Otoneurology, Department of Otorhinolaryngology, Complejo Hospitalario Badajoz, Badajoz, Spain; ^19^Unit of Chronic Inflammatory Diseases, Institute of Environmental Medicine, Karolinska Institutet, Stockholm, Sweden; ^20^Luxembourg Centre for System Biomedicine (LCSB), Universite du Luxembourg, Belval, Luxembourg

**Keywords:** TNFRSF12A, NFKB1, TWEAK/Fn14 pathway, NF-κB signaling, vertigo, sensorineural hearing loss, Meniere’s disease

## Abstract

Meniere’s disease (MD) is a rare disorder characterized by episodic vertigo, sensorineural hearing loss, tinnitus, and aural fullness. It is associated with a fluid imbalance between the secretion of endolymph in the cochlear duct and its reabsorption into the subarachnoid space, leading to an accumulation of endolymph in the inner ear. Epidemiological evidence, including familial aggregation, indicates a genetic contribution and a consistent association with autoimmune diseases (AD). We conducted a case–control study in two phases using an immune genotyping array in a total of 420 patients with bilateral MD and 1,630 controls. We have identified the first locus, at 6p21.33, suggesting an association with bilateral MD [meta-analysis leading signal rs4947296, OR = 2.089 (1.661–2.627); *p* = 1.39 × 10^−09^]. Gene expression profiles of homozygous genotype-selected peripheral blood mononuclear cells (PBMCs) demonstrated that this region is a *trans*-expression quantitative trait locus (eQTL) in PBMCs. Signaling analysis predicted several tumor necrosis factor-related pathways, the TWEAK/Fn14 pathway being the top candidate (*p* = 2.42 × 10^−11^). This pathway is involved in the modulation of inflammation in several human AD, including multiple sclerosis, systemic lupus erythematosus, or rheumatoid arthritis. *In vitro* studies with genotype-selected lymphoblastoid cells from patients with MD suggest that this trans-eQTL may regulate cellular proliferation in lymphoid cells through the TWEAK/Fn14 pathway by increasing the translation of NF-κB. Taken together; these findings suggest that the carriers of the risk genotype may develop an NF-κB-mediated inflammatory response in MD.

## Introduction

Meniere’s disease [MD (MIM 156000)] is an inner ear syndrome characterized by recurrent attacks of vertigo associated with concurrent ipsilateral aural symptoms, such as fluctuating sensorineural hearing loss (SNHL), tinnitus, or aural pressure (1, 2). MD is associated with a fluid imbalance between the secretion of endolymph in the cochlear duct and the reabsorption into the subarachnoid space, leading to an accumulation of endolymph termed endolymphatic hydrops (3), but the underlying molecular mechanism remains unknown.

Epidemiological evidences support a genetic contribution in MD including: (a) a higher prevalence of MD in Caucasians over other ethnicities (4) and (b) familial clustering, as familial MD occurs in 6–10% of patients with MD in European and Asian-descent populations, respectively, and it has a high sibling recurrence risk ratio (λ_s_ = 24–45) (5, 6). Early case–control studies in small series using candidate genes suggested an association with HLA class II genes in different populations (7); however these studies have not been replicated (8). By contrast, a genomic approach using whole-exome sequencing in families with autosomal-dominant MD and autoimmune background has identified rare variants with potential pathogenic effects in the *FAM136A, DTNA, PRKCB, DPT*, and *SEMA3D* genes (9–11). Although these candidate genes for familial MD should be confirmed in sporadic and more families with MD, they start to anticipate genetic heterogeneity.

Different studies have described a MD association with several autoimmune diseases (AD), such as rheumatoid arthritis, systemic lupus erythematous (SLE), or psoriasis (12, 13). Based on the results of proteomic studies performed in small series of patients, autoimmunity has been proposed as a potential cause of MD (14, 15). However, elevated immune complexes were only found in 7% of patients with MD (16), and there is no consistent immunological biomarker for the diagnosis of MD. Therefore, the evidence to support the hypothesis of autoimmunity is limited. The TWEAK/Fn14 pathway is involved in the modulation of inflammation in several chronic AD, including multiple sclerosis, SLE, rheumatoid arthritis, or ulcerative colitis (17). However, this pathway has not been investigated in SNHL or MD.

Nuclear factor kappa B (NF-κB) is a family of transcription factors, which regulate immune and inflammatory responses. In the latent state, NF-κB is inhibited in the cytosol by IκB (inhibitor of NF-κB) proteins. Upon stimulation of innate immune receptors such as cytokines or toll-like receptors, a series of membrane proximal events lead to the activation of IκB kinases (IKK). Phosphorylation of IκBs releases NF-κB, which translocates to the nucleus to regulate gene transcription (18).

Bilateral involvement in MD (BMD) may occur in 20–47% of patients after 10 years of follow-up (19). Most patients begin with vertigo and hearing loss in one ear, and hearing loss can appear in the second ear several years later, but a significant number of individuals show simultaneous SNHL. Autoimmune inner ear disease (AIED) is a rare disorder defined by recurrent episodes of bilateral SNHL progressing over a period of several weeks or months (20). Vestibular symptoms may be present in 50% of patients and systemic autoimmune disease coexists in 30% of patients (21). This audiovestibular phenotype overlaps with BMD and it may not be possible to distinguish AIED and MD. In some cases, AIED may begin as sudden unilateral SNHL involving rapidly the second ear. Although the mechanism of AIED is not well understood, these patients show elevated levels of proinflammatory cytokines, including IL-1β and TNFα (22), and may respond to steroid therapy or anakinra (23). Furthermore, autoimmune endolymphatic hydrops was described in patients with Cogan syndrome and polyarteritis nodosa and it was found in 50% of patients with AIED.

The aim of this study was to identify susceptibility loci using the Immunochip genotyping array to define a subset of patients with MD, which may have an autoimmune dysfunction. Here, we found a locus in 6p21.33 and we demonstrated that it regulates gene expression in the tumor necrosis factor (TNF)-like weak inducer of apoptosis (TWEAK)/Fn14 pathway and induces translation of NF-κB in lymphoid cells.

## Materials and Methods

### Ethics Approval Statement

The study protocol PI13/1242, with reference 01-2014, was approved by the ethic Committee for clinical research of all the recruiting centers. All participants gave written informed consent. The work was performed according to the principles of the Declaration of Helsinki of 1975 (as revised in 2013) (24).

### Case Definition and Sample Population

Meniere’s disease cases were diagnosed according to the clinical guidelines defined by the Committee on Hearing and Equilibrium of the American Academy of Otolaryngology Head and Neck Surgery (AAO-HNS) (25). All familial cases were excluded.

The initial cohort consisted of 681 cases of MD (492 unilateral and 189 bilateral SNHL) and 735 unrelated controls. The replication cohort was drawn from an independent group of 240 bilateral cases and 895 Iberian controls of European ancestry. The samples included in the discovery cohort were partially overlapped with a preliminary study previously published (26).

The diagnosis protocol included a complete neuro-otological evaluation including otoscopy, a pure-tone audiometry, nystagmus examination and caloric testing, and a brain MRI to exclude other possible causes of neurological symptoms. Patients were monitored with serial audiograms and the following clinical variables were studied in our series: gender, age, hearing stage, duration of the disease, bilateral SNHL, age of onset, type of headache, history of autoimmune disease, smoking, Tumarkin crisis, and the functional scale of the AAO-HNS. Hearing stage was calculated with the audiogram obtained the day of inclusion for each patient with definite MD and was defined as the mean of four-tone average of 0.5, 1, 2, and 3 kHz according to the AAO-HNS criteria: stage 1, ≤25 dB HL; stage 2, 26–40 dB HL; stage 3, 41–70 dB HL; and stage 4, >70 dB HL.

### DNA and RNA Extraction

DNA was isolated from peripheral blood using the QIAamp DNA Mini Kit (Qiagen, Venlo, Netherlands), according to the manufacturer’s instructions. The concentration of genomic DNA was measured using the Qubit dsDNA BR Assay Kit (Invitrogen, ThermoFisher Scientific, Waltham, MA, USA) and concentrations were standardized to 50 ng/mL for genotyping, the quality was determined by Nanodrop 2,000 C (ThermoFisher Scientific, Waltham, MA, USA).

Total RNA was obtained from peripheral blood mononuclear cells (PBMC) using the High Pure RNA Isolation Kit (Hoffmann-La Roche, Basel, Switzerland) following the manufacturer’s protocols. The quantity and quality of total RNA were determined using the RNA Nano assay on the Agilent 2100 Bioanalyzer (Agilent Technologies, Waldbronn, Germany).

### Genotyping and Quality Controls (QC)

DNA samples were genotyped by the Immunochip, a custom genotyping array which includes loci previously associated with 12 autoimmune disorders (27). Clusters were manually inspected and verified, and SNPs with poor clustering quality metrics were removed (call frequency <0.98, cluster separation <0.4, and GenCall scores <0.15). Further, the SNPs that did not meet the following criteria were excluded: minor allele frequency (MAF) <5%, Hardy–Weinberg equilibrium <10^−4^ in controls, non-random differential missing data rate test between cases and controls <10^−5^, and missing-genotype rate <0.5%. All markers in chromosome X were also excluded. After QC, 96,899 single nucleotide variants (SNVs) remained with a MAF >5% for statistical analysis.

Samples with a genotype success rate of <90% and increased heterozygosity rate (<0.18 and >0.45) were excluded from the analysis. Finally, genetic outliers determined by principal-component analysis (PCA) were removed from the analysis (>3 SD around the mean).

The genotyping of the replication cohort was performed with the TaqMan SNP assay in an ABI 7500 Fast Real-Time PCR System (Life Techonologies, Carlsbad, CA, USA). The alleles were determined using the SDS 2.2.1 software (Applied Biosystems, Foster City, CA, USA). We used PCA to identify population substructure. Furthermore, a representative sample of SNVs genotyped by the Immunochip was validated also by Taqman assays in 165 individuals. The correlation coefficient between both methods was 98%. Genotype calling was performed in all samples with the Genotyping Module (v1.8.4) of the Genome Studio Data Analysis Software. NCBI Build 36 (hg18) mapping was used (Illumina manifest file Immuno_BeadChip_11419691_B.bpm). Data were converted into the human Build hg38 using.^1^

Quality controls were performed, for each set of samples and SNVs separately, using Genome Studio Data Analysis Software and PLINK software (version 1.07) (28). After all QC, 189 patients with bilateral SNHL and 735 controls remained for further statistical analyses. We have evaluated the association between each SNV and patients with unilateral or bilateral MD.

### Gene Expression Assay in PBMCs

Peripheral blood mononuclear cells were isolated from peripheral blood of patients with the main genotypes of SNVs rs4947296 by Ficoll gradients (Biowest, Nuaillé, France). After RNA extraction, gene expression levels were quantified using the Illumina HumanHT-12 v4 Expression BeadChip (Illumina Inc., San Diego, CA, USA). Probe intensity data were analyzed using Illumina’s GenomeStudio software (Gene Expression Module) to determine the gene expression levels according to negative control probes for background correction and quantile normalization using negative and positive control probes. Probes with detection *p*-values < 0.05 in less than 10% of samples were filtered, and replicated genes were removed using the median value. Differential expression analysis between samples was performed using the R limma package. Furthermore, we evaluated if the expression of the genes located at <1 Mb distance from the locus and the MHC region were affected by rs4947296 (*p* < 0.05).

Data from the expression array can be accessed at the Gene Expression Omnibus under accession number GSE77865.

### Bioinformatics Analysis

Signaling pathway analysis was performed using Ingenuity Pathways Analysis (IPA^®^, Qiagen, Venlo, Netherlands^2^) software. Core analysis tool was executed using the differentially expressed gene (DEG) with an adjusted *p*-value cutoff of 0.001. The most significant pathway was the “TWEAK Signaling pathway.” Pathway enrichment analysis was performed with MetaCore (GeneGo^3^) (29), using the DEG with the enrichment *p*-value cutoff of 0.001. The three enriched canonical pathways “apoptosis and survival Apoptotic TNF-family pathways,” “signal transduction NF-κB activation pathways,” and “apoptosis and survival Anti-apoptotic TNFs-NF-κB-Bcl-2 pathway” were retrieved and the shortest paths from Fn14 (TNFRSF12A) to NF-κB genes were extracted. The shortest paths were visualized in Cytoscape ver. 2.7.0 (30).

### Cell Culture

Peripheral blood mononuclear cells were seeded at a density of 5 × 10^6^ cells/mL in RPMI 1640 (Thermo Fisher Scientific, Waltham, MA, USA) containing 20% Fetal Bovine Serum (FBS, Biowest, Nuaillé, France) and Epstein–Barr virus at 1:1 ratio was added to generate lymphoblasts. Cells were placed in an incubator maintained at 37°C with 7% CO_2_ and cultured in RPMI 1640 supplemented with 10% FBS, non-essential amino acids, and sodium pyruvate.

Cell viability and proliferation assays were performed in lymphoblastoid cell lines (LCL) to investigate the effect of the rs4947296 homozygous conditional genotypes. Five thousand cells were plated in 96-well plates and incubated at different TWEAK (PeproTech, London, UK) concentrations to examine the effect over both cell lines (0, 50, 100, 250, and 500 ng/mL) (31). Proliferation rate was measured at 24, 48, and 72 h. At each time point, 20 μL of PrestoBlue™ (Life Technologies, Carlsbad, CA, USA) was added to each well and cultured at 37°C for 4 h. After that, the absorbance of the supernatant was measured at 570 nm in a Tecan Infinite Nanoquant M200 Pro absorbance microplate reader. Blank controls were performed for each measure using medium and PrestoBlue™ (Life Technologies, Carlsbad, CA, USA). Cell viability assay was performed using Trypan blue staining (Thermo Fisher Scientific, Waltham, MA, USA). The size of the clusters was measured using the area (μm^2^) of 200 clusters for each genotype by ImageJ software (ImageJ, U. S. National Institutes of Health).

### Quantitative RT-PCR (qPCR)

Quantitative RT-PCR was performed using the Brilliant III Ultra-Fast SYBR^®^ Green qPCR Master Mix (Agilent Technologies, Santa Clara, CA, USA) and an ABI 7900 HT Fast real-time PCR Systems (Life Technologies, ThermoFisher Scientific, Waltham, MA, USA) using primers listed in Table 1. Hypoxanthine phosphoribosyltransferase 1 was used as housekeeping gene. Technical triplicates were performed to reduce experimental errors. The fold change for each gene was obtained using the comparative CT method (32). Statistical analyses were performed using Student’s *t*-test. A *p* value < 0.05 was considered statistically significant.

**Table 1:** Primer sequences for quantitative RT-PCR.

GENE	Forward primers	Reverse primers
NFKB1	5′-GAAGCACGAATGACAGAGGC-3′	5′-GCTTGGCGGATTAGCTCTTTT-3′
TNFRSF12A	5′-CTGGCTCCAGAACAGAAAGG-3′	5′-GGGCCTAGTGTCAAGTCTGC-3′
LFA-1	5′-TTGGGGTTTGAAGAAGTCTCAG-3′	5′-GTGCCTCCCATTGAAGATGT-3′
ICAM-1	5′-GATTCTGACGAAGCCAGAGG-3′	5′-CCGGGTCTGGTTCTTGTGTA-3′
Hypoxanthine phosphoribosyltransferase 1	5′-TGACACTGGCAAAACAATGCA-3′	5′-GGTCCTTTTCACCAGCAAGCT-3′
FOS	5′-GGGGCAAGGTGGAACAGTTAT-3′	5′-CCGCTTGGAGTGTATCAGTCA-3′
BIRC3	5′-AAGCTACCTCTCAGCCTACTTT-3′	5′-CCACTGTTTTCTGTACCCGGA-3′
FADD	5′-GTGGCTGACCTGGTACAAGAG-3′	5′-GGTAGATGCGTCTGAGTTCCAT-3′
NFKBIE	5′-TCTGGCATTGAGTCTCTGCG-3′	5′-AGGAGCCATAGGTGGAATCAG-3′
CASP3	5′- GTACAGATGTCGATGCAGCAA-3′	5′- GCACACAAACAAAACTGCTCC-3′
CASP6	5′- CGATGTGCCAGTCATTCCTTT-3′	5′- GCTGCATCCACCTCAGTTATG-3′
CASP9	5′- CAGAGATTCGCAAACCAGAGG-3′	5′- CACCGACATCACCAAATCCTC-3′
APAF1	5′- GCCCTGCTCATCTGATTCATG-3′	5′- TCTCACTGACTGCACAATCCT-3′
CYCS	5′- AAGACTGGGCCAAATCTCCAT-3′	5′- TCTGCCCTTTCTTCCTTCTTCT-3′
IKBKG	5′- GATCTCAAACAGCAGCTCCAG-3′	5′- AGTCCGCCTTGTAGATATCCG-3′

### Western Blot

Protein extraction was carried out by acetone precipitation (33). Protein concentration was determined by Bradford protein assay (Bio-Rad Laboratories, Hercules, CA, USA) and total protein was stored at −80°C. Sixty micrograms of total proteins were separated by molecular weight in a poliacrilamyde gel [Criterion™ TGX™ Precast Gels (Bio-Rad Laboratories, Hercules, CA, USA)] and transferred to a Trans-Blot^®^ Turbo™ Midi PVDF membrane by Trans-Blot^®^ Turbo™ Transfer System (Bio-Rad Laboratories, Hercules, CA, USA). The membrane was incubated with primary antibody against NF-κB p105/p50 (Abcam, Cambridge, UK; #ab7971, 1:400) overnight at 4°C and a chicken polyclonal antibody against GAPDH (EMD Millipore, #AB2302, 1:1,000). Then, the membrane was incubated with secondary antibodies for 1 h at room temperature. A goat anti-rabbit (R&D Systems, #HAF008, 1:3,000) and a rabbit anti-chicken (Sigma-Aldrich, #A9046-1ML, 1:9,000) were used, respectively. After that, the membrane was developed using Clarity™ Western ECL Substrate (Bio-Rad Laboratories, Hercules, CA, USA) and the images were obtained using the ImageQuant LAS4000 (GE Healthcare Life Science). ImageJ software (NIH, USA) was used for the quantification.

### Confocal Image Analysis of Whole Mount LCLs

Selected LCLs were obtained from patients with MD according to the genotype. LCLs undergoing TWEAK treatment (250 ng/mL) for 48 h were fixed using fresh methanol: DMSO (4:1) and stored at −20°C until used. LCLs were then rehydrated, blocked, stained, and mounted as previously described (34). Primary antibodies were used as follows: a mouse monoclonal antibody against Fn14 (Santa Cruz Biotechnology, Dallas, TX, USA; #sc-56250, 1:50) and a rabbit polyclonal antibody against NF-κB p105/p50 (Abcam, Cambridge, UK; #ab7971, 1:50). As secondary antibodies, we used Alexa-555-conjugated goat anti-mouse (Life Technologies, Carlsbad, CA, USA; #A-21422, 1:500) and Alexa-633-conjugated goat anti-rabbit (Life Technologies, Carlsbad, CA, USA; #A-21071, 1:500), respectively. For nuclei staining, we used Hoechst 3342 (Life Technologies, Carlsbad, CA, USA; #H1399, 1:1,000). A laser scanning confocal microscope LSM 710 (Carl Zeiss, Oberköchen, Germany) was used for image collection and the Zeiss browser software program ZEN black edition was used to acquire and export the data. All images were taken with the same laser intensity settings on the microscope and final image processing and labeling were performed with ImageJ.

### NF-κB p65 Phosphorylation Assay

Lymphoblastoid cell lines according to each genotype were plated with a density of 1 × 10^6^cells/mL and treated with TWEAK (250 ng/mL) during 48 h at 37°C. After that time, cells were centrifuged and resuspended in an appropriate volume of HBSS containing 5% FBS (Biowest, Nuaillé, France). Cells were then lysed with Cell Lysis Buffer 5× from the NF-κB p65 (Total/phospho) Multispecies InstantOne™ ELISA Kit (Thermo Fisher Scientific, Waltham, MA, USA) and manufacturer’s protocol was followed.

### Statistical Analysis

We performed a descriptive statistical analysis for clinical variables, using SPSS software v.22 (SPSS Inc., Chicago, IL, USA). Data are shown as means with their SD. Quantitative variables were compared using Student’s unpaired *t*-test. Qualitative variables were compared using crosstabs and Fisher’s exact test. Nominal *p*-values using a 5% level to determine significance are reported. Allelic and genotypic frequencies were compared between patients and controls by logistic regression test and calculating the odds ratios (OR) and 95% confidence intervals using PLINK (version 1.07). Genotypes were imputed and implemented in IMPUTEv2 using the 1,000 Genomes Phase 3 integrated reference panel according to a previously described method (35).

Potential interactions between associated loci were also tested using the association module in PLINK v1.07. Logistic regression analyses were used to estimate the genotype-specific effects of the risk alleles.

We selected SNVs for the replication study based on the results of the discovery phase and the meta-analysis was performed by SPSS. The functional evaluation of each SNP located in candidate loci was performed *in silico* using HaploReg,^4^ which provides linkage disequilibrium information (*r*^2^ and *D*′ measurements) and it allows us to define haplotype blocks in each chromosome used (36). Moreover, we used seeQTL^5^ and RegulomeDB^6^ to annotate regulatory variants of the noncoding genome such as enhancers, transcription factors binding sites, their conservation across mammals and their potential effects on regulatory motifs (37, 38).

Clinical variables were compared between patients with unilateral and BMD by unpaired *t* test for quantitative variables and χ^2^ test for qualitative variables. *P* < 0.05 was considered statistically significant.

## Results

### Bilateral MD Is Associated with a Locus in the Classical Class I Subregion of the MHC

Table 2 compares the clinical features of 1,451 patients with uni and bilateral SNHL in MD. Patients with bilateral SNHL had a longer duration of the disease (*p* = 1.5 × 10^−6^), worse hearing loss at diagnosis (*p* = 2.5 × 10^−4^), worse hearing stage (*p* = 2 × 10^−6^), higher frequency of AD (*p* = 4 × 10^−3^), and higher frequency of migraine (*p* = 6 × 10^−3^).

**Table 2:** Clinical features of patients with sporadic Meniere’s disease.

Variables	Bilateral (*n* = 420)	Unilateral (*n* = 1,031)	*p*-Value
Age of onset, mean (SD)	45.23 (13.6)	47.24 (12.1)	0.263
Gender (% women)	59.3	57.5	0.598
Time course (years), mean (SD)	15.16 (9.1)	10.35 (±7.7)	**1.50 × 10^−06^**
Affected ear (%)		Right (50.9)	
Hearing loss at diagnosis, mean (SD)	56.96 (16.7)	48.66 (19.1)	**2.47 × 10^−04^**
Migraine, *n* (%)	70 (18.4)	131 (12.8)	**0.006**
History of autoimmune disease, *n* (%)	63 (19.2)	119 (12.8)	**0.004**
Smoking, *n* (%)	81 (21.3)	254 (24.3)	0.422

**Hearing stage, ***n*** (%)**			
1	7 (2.0)	117 (12.2)	**5.00 × 10^−06^**
2	52 (14.9)	232 (24.2)	
3	178 (51.0)	471 (49.1)	
4	112 (32.1)	140 (14.6)	
Hearing stage, mean (SD)	3.13 (0.7)	2.66 (0.8)	**2.00 × 10^−06^**
Turmakin crisis, *n* (%)	85 (29.5)	126 (16.9)	**8.00 × 10^−06^**

**Functional scale, ***n*** (%)**			
1	37 (12.6)	167 (12.6)	**0.008**
2	91 (31.0)	332 (37.1)	
3	74 (25.2)	185 (20.7)	
4	46 (15.6)	126 (14.1)	
5	37 (12.6)	71 (7.9)	
6	8 (2.7)	13 (2.7)	

*Numbers in bold represent significant *p*-values (*p* < 0.05)*.

**Figure 1:**
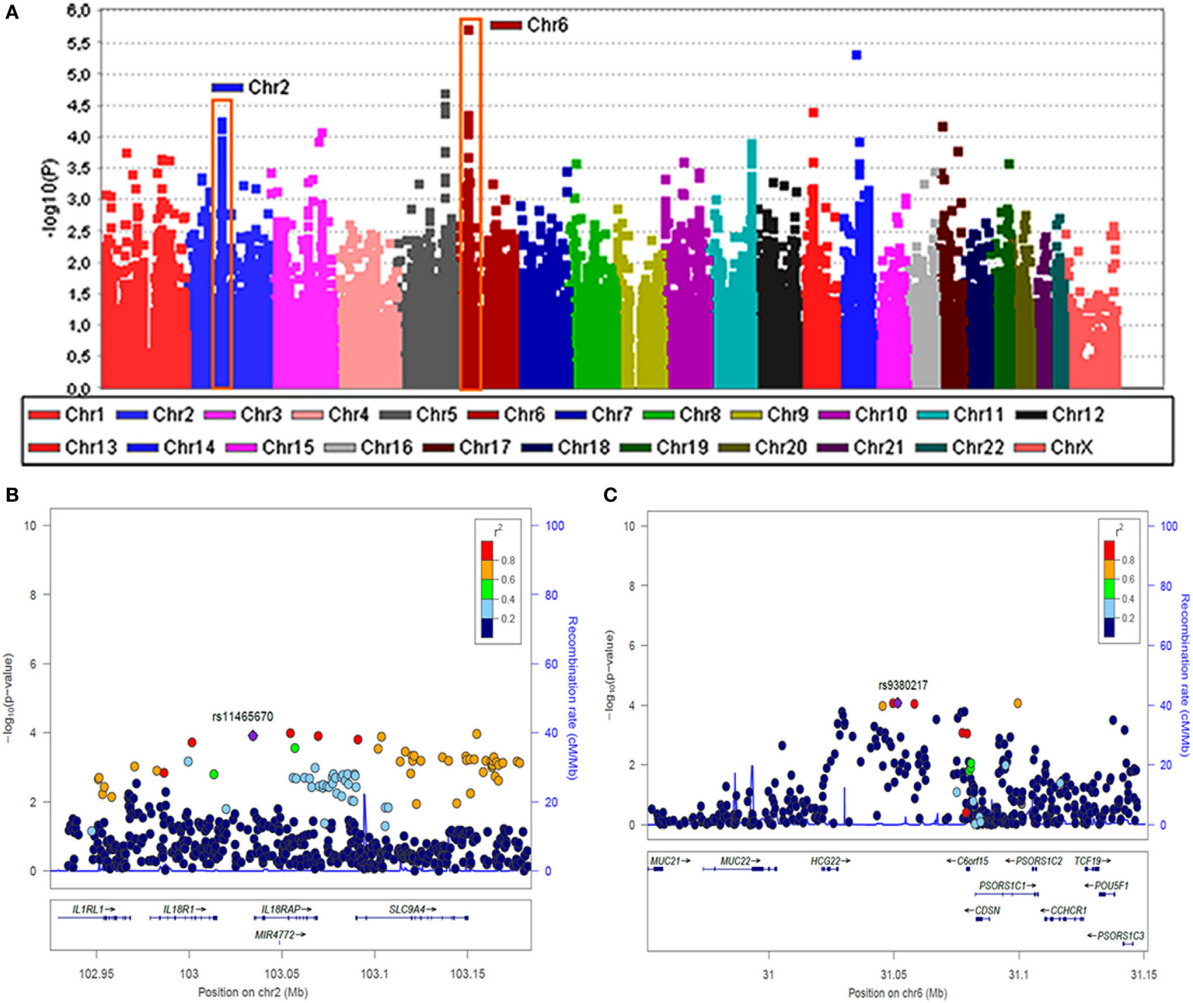
Two loci associated with bilateral sensorineural hearing loss (SNHL). **(A)** Manhattan plot for association study findings from Immunochip genotyped bilateral cases and controls. **(B)** Association area at the region on chromosome 2 and **(C)** association area at the region on chromosome 6. Both **(B,C)** −logP values of single nucleotide variants (SNVs) associated with bilateral SNHL are shown on the left *y*-axis and the recombination rates expressed in centimorgans (cM) per Mb, are shown on the right *y*-axis. Positions in Mb are on the *x*-axis (NCBI Build GRCh38). Linkage disequilibrium for each SNV with the top SNV, displayed as a large purple diamond, is indicated by its color. The plots were drawn using LocusZoom tool (http://locuszoom.sph.umich.edu/locuszoom/).

Although no significant association was found in patients with unilateral MD, two genomic regions at chromosome 2 and 6 reached confirmatory significance (*p*-values < 10^−6^) in the subset of patients with BMD (Figure 1). To perform the replication, we selected representative TagSNVs, according to the results of the discovery phase in both regions (Table 3). The meta-analysis confirmed a suggestive significant association with a locus in the classical class I subregion of the MHC ~9 kb at 6p21.33 (31,081,878–31,090,401), being the leading SNV rs4947296; OR = 2.089 (1.661–2.627); *p* = 1.39 × 10^−09^. Conditional regression analysis showed no independent associated signals in chromosome 6, and the association between this locus and BMD remained robust when it was adjusted to any variant in the region. So, according to rs4947296, we defined the homozygous risk genotype as CC and the protective genotype as TT for further studies.

**Table 3:** Meta-analysis of loci associated with bilateral Meniere’s disease.

SNV					Phase 1 (*n* = 189 cases; 735 controls)				Phase 2 (*n* = 240 cases; 895 controls)				Meta-analysis (*n* = 429 cases; 1,630 controls)			
Chr.	Pos.	Rs	Ref.	Alt.	RAF_C	RAF_N	OR (95%)	*p*-Value	RAF_C	RAF_N	OR (95%)	*p*-Value	RAF_C	RAF_N	OR (95%)	*p*-Value
2	102351615	rs4988957	C	T	0.414	0.358	1.27 (0.99–1.62)	5.24E−02	0.381	0.355	1.07 (0.93–1.23)	1.88E−01	0.406	0.351	1.16 (1.05–1.83)	2.91E−03
2	102417980	rs11465670	T	C	0.156	0.093	1.81 (1.29–2.54)	5.02E−04	0.087	0.083	1.04 (0.73–1.49)	4.41E−01	0.121	0.087	1.38 (1.10–1.73)	4.52E−03
2	102460685	rs4851589	A	G	0.337	0.279	1.32 (1.02–1.69)	3.35E−02	0.247	0.258	0.96 (0.79–1.16)	3.61E−01	0.299	0.267	1.12 (0.99–1.27)	4.53E−02
6	30814225	rs886424	C	T	0.102	0.051	2.11 (1.39–3.21)	3.55E−04	0.082	0.071	1.14 (0.81–1.62)	2.55E−01	0.095	0.067	1.41 (1.09–1.83)	7.73E−03
6	31083776	rs9380217	C	T	0.159	0.069	2.55 (1.81–3.58)	3.40E−07	0.108	0.078	1.43 (1.00–2.06)	5.52E−02	0.132	0.074	1.854 (1.465–2.347)	2.02E−07
6	31090401	**rs4947296**	T	C	0.164	0.067	2.72 (1.93–3.82)	3.15E−08	0.108	0.078	1.432 (1.024–2.004)	3.52E−02	**0.142**	**0.073**	**2.089 (1.661–2.627)**	**1.39E−09**
6	32082981	rs1150754	C	T	0.117	0.061	2.04 (1.38–3.01)	2.66E−04	0.089	0.067	1.16 (0.81–1.65)	2.35E−01	0.096	0.071	1.36 (1.05–1.77)	1.34E−02

*Chr., chromosome; SNV, single nucleotide variant; Pos., genomic position; Rs, SNV identifier; Ref., reference; Alt., alternative allele on human genome reference GRCh38; RAF_C, risk allelic frequency in cases; RAF_N, risk allelic frequency in controls; OR, odds ratio*.

### The rs4947296 Regulates Gene Expression in the TWEAK/Fn14 Pathway in PBMCs

**Figure 2:**
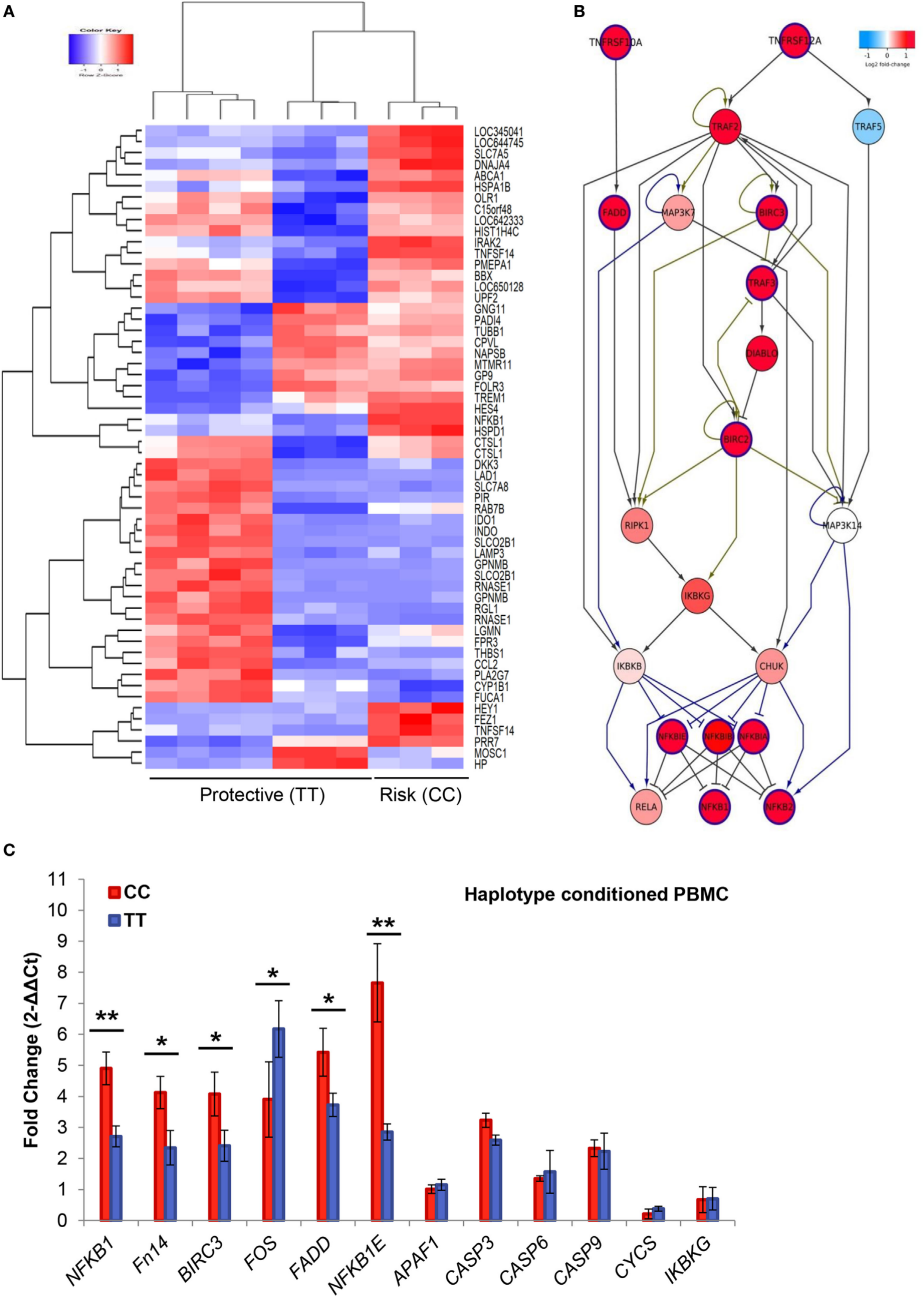
Gene expression in peripheral blood mononuclear cells. **(A)** Heatmap of 973 differentially expressed genes (DEG). Samples and genes (columns and rows, respectively) are reordered on the basis of the normalized expression value and give rise to groups of genes and samples with similar expression levels, according to the color key. The samples (column) were clustered into two groups according to rs4947296: three individuals with CC genotype (risk) and seven individuals with TT genotype (protective). **(B)** The shortest path from Fn14 (*TNFRSF12A*) to *NFKB* genes. DEG in mononuclear cells (adjusted *p* < 0.001), according to the homozygous genotype, were used to predict involved pathways. The network was retrieved from three MetaCore pathways [“apoptosis and survival Apoptotic tumor necrosis factor (TNF)-family pathways,” “signal transduction NF-κB activation pathways,” and “apoptosis and survival Anti-apoptotic TNFs-NF-κB-Bcl-2 pathway”] enriched in our pathway enrichment analysis. Log fold change is color-coded, where red nodes indicate upregulated genes, whereas blue nodes indicate downregulated genes. Activation interactions are indicated by arrow heads, whereas inhibitory interactions are indicated by blunted heads. Black edges indicate physical binding interaction, purple edges indicate phosphorylation, and brown edges indicate ubiquitination. Genes with thick purple margin are DEG. **(C)** Quantitative RT-PCR validation of genes involved in the TWEAK/Fn14 pathway (*NFKB1, Fn14, BIRC3, FADD, NFKBIE, FOS, APAF1, CASP3, CASP6, CASP9, CYCS*, and *IKBKG*) (*p < 0.03, **p < 0.0005).

We compared the gene expression profile of PBMCs from 10 individuals according to the rs4947296 genotype (CC vs. TT). We demonstrated that this region is an expression quantitative trait locus (eQTL) in mononuclear cells, showing significant differences in the expression levels of 973 genes (adjusted *p* < 0.001, Figure 2A; Table S1 in Supplementary Material). Selecting those genes showing a differential expression according to the genotype, pathway analysis performed by IPA^®^ software, predicted the activation of several candidate pathways associated with TNF (Table S2 in Supplementary Material). The TWEAK/Fn14 pathway showed 31 differentially expressed genes (DEG, 88.5%; *p* = 2.42 × 10^−11^). Moreover, the eQTL was also associated with the activation of the Death Receptor signaling pathway with 64 DEG (68.8%; *p* = 8.45 × 10^−11^); TNFR2 signaling pathway with 26 DEG (86.6%; *p* = 2.97 × 10^−9^), and TNRF1 signaling pathway with 37 DEG (74%; *p* = 6.69 × 10^−9^).

The enrichment analysis of canonical pathways in MetaCore (adjusted *p* < 0.001) also resulted in several TNF-related pathways for apoptosis and inflammation that contained TWEAK/Fn14 sub-pathway (Table S3 in Supplementary Material). To gain insight into the possible molecular interactions that mediate TWEAK signaling to NF-κB, we extracted three enriched canonical pathways “apoptosis and survival Apoptotic TNF-family pathways,” “signal transduction NF-κB activation pathways,” and “apoptosis and survival Anti-apoptotic TNFs-NF-κB-Bcl-2 pathway” from MetaCore. The shortest paths from Fn14 (*TNFRSF12A*) to *NFKB1* genes were extracted (Figure 2B) and visualized in Cytoscape v.2.7.0 (30). These shortest paths involved several DEG, including *BIRC3* and *NFKBIE*. Although *FADD* was not among these shortest paths, it is along the *TNFRSF10A*-induced path that feeds into the TWEAK/Fn14 path, suggesting its complementary role in the TWEAK/Fn14 signaling.

We also validated the gene expression profile of *NFKB1, TNFRSF12A, BIRC3, FADD, NFKBIE, FOS, CASP3, CASP6, APAF1, IKBKG, CYCS*, and *CASP9* genes in mononuclear cells from patients by qPCR, according to the selected genotypes (Figure 2C).

### TWEAK Induces Cluster Formation and Proliferation in Selected Lymphoblasts

**Figure 3:**
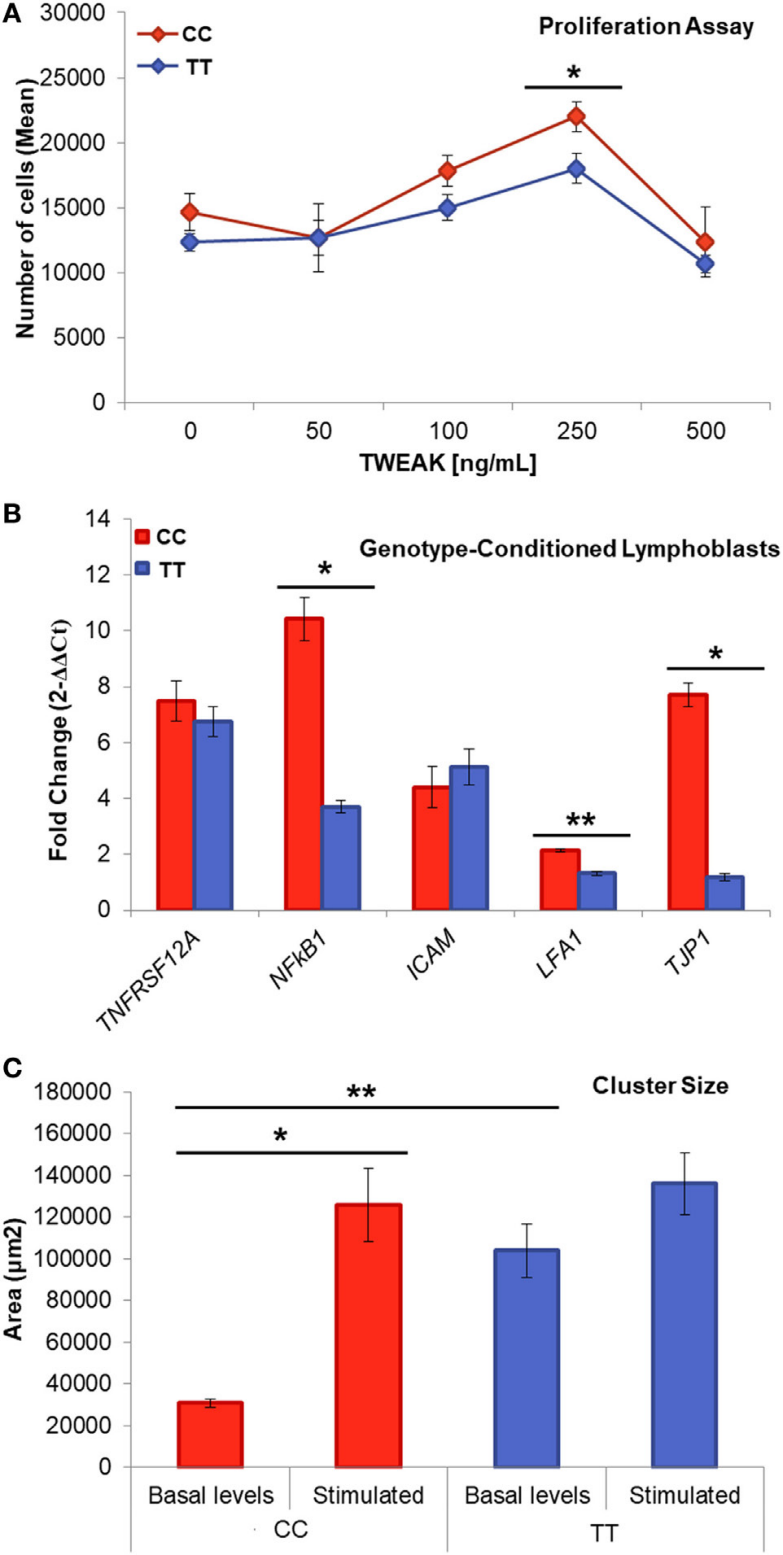
Genotype-conditioned lymphoblastoid cell lines (LCL) for rs4947296. **(A)** Proliferation assay of homozygous LCL treated with tumor necrosis factor-like weak inducer of apoptosis (TWEAK; 0, 50, 100, 250, and 500 ng/mL) and measured using PrestoBlue™ (*p < 0.05). Comparisons between groups were achieved using a two-sided Student’s *t*-test. **(B)** Relative gene expression according to the genotype, after treatment with 250 ng/mL of TWEAK, *p*-values: *TNFRSF12A* = 0.44; *NFKB1* = 1.36 × 10^−4^; *ICAM* = 0.91; *LFA-1* = 5 × 10^−6^; *TJP1* = 3.2 × 10^−5^ (*p < 1 × 10^−4^, **p < 5 × 10^−6^) **(C)** Cluster size within LCLs with and without stimulation (**p = 5 × 10^−7^, *p = 0.02). CC, risk genotype; TT, protective genotype.

Lymphoblastoid cell lines proliferate forming clusters with rosette morphology due to the expression of adhesion molecules such as LFA-1 (leukocyte function antigen 1 encoded by *ITGB2* gene) also known as CD11a/CD18 and its ligand, ICAM-1 (intercellular adhesion molecule 1, CD54) in the plasma membrane. Interestingly, the size of the clusters showed significant differences according to the genotype, being smaller for the risk genotype (CC: 30,968.88 ± 1,960.45 μm^2^; TT: 103,921.33 ± 12,720.92 nm, *p* = 5 × 10^−7^) (Figure 3A).

When we treated the cells with TWEAK at a concentration of 250 ng/mL, we observed a marked increase in the size of the risk genotype clusters, which was not observed in the protective genotype (CC: 125,609.84 ± 17,502.21 μm^2^, *p* = 2 × 10^−6^; TT: 136,132.42 ± 14,785.38 μm^2^, *p* = 0.02). This experiment shows that TWEAK induces a significant aggregation of LCLs in the carriers of the risk genotype.

Next, we compared the effect of TWEAK in the proliferation of selected LCLs. So, 250 ng/mL TWEAK increased the proliferation rate after 48 h in both cell lines (CC *p* = 0.017, TT, *p* = 0.013; Figure 3C). This effect suggests the activation of the non-canonical NF-κB signaling *via* Fn14 receptor that we confirmed showing an increase expression of *TNFRSF12A* and *NFKB1* genes (Figure 3B).

To investigate if the difference in the cluster formation was related with the differential expression of cell adhesion molecule genes, we measured the mRNA levels of three cell surface markers in LCLs: the integrin LFA-1 and the adhesion molecule ICAM which binds to integrins, as well as tight-junction protein ZO-1 (*TJP1 gene*), which interacts directly with actin. We found a significant increase in the expression of *ITGB2* (*p* = 5 × 10^−6^; Figure 3B) and in *TJP1* (*p* = 3.2 × 10^−5^) in the risk genotype, which was not observed in the protective genotype. The differences in clusters size, *ITGB2* and *TJP1* expression, according to the genotype, are consistent with the hypothesis that this eQTL could regulate lymphoblasts adhesion and proliferation.

### The rs4947296 May Regulate Phosphorylation in NF-κB p65 Subunit on Serine 536 in Lymphoblasts

**Figure 4:**
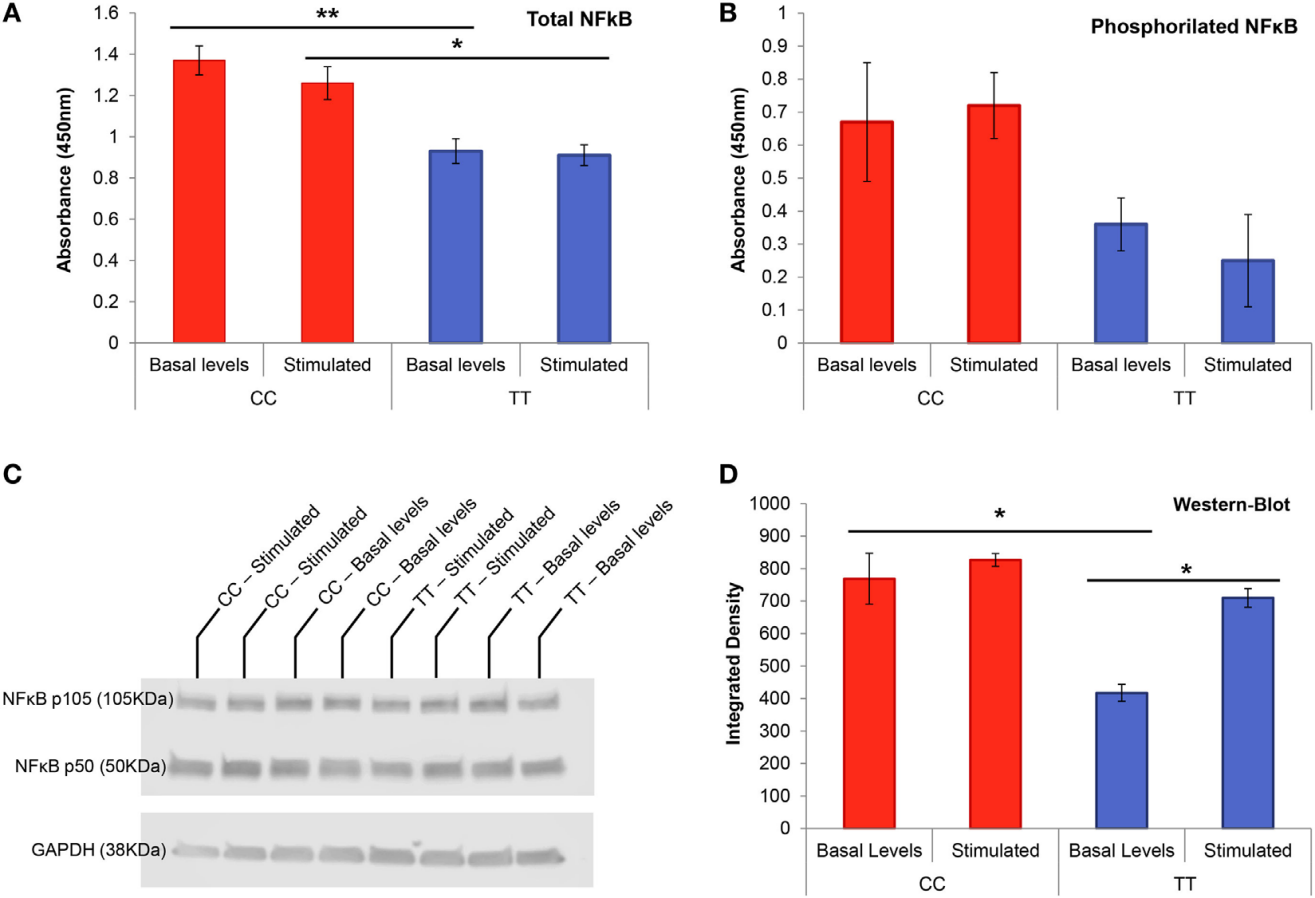
NF-κB transcription factor protein studies in genotype-conditioned lymphoblastoid cell lines (LCLs). **(A)** Total NF-κB ELISA performed in homozygous LCL for rs4947296 at basal levels and after stimulation with TWEAK at 250 ng/mL (*p < 0.02, *p < 0.006). **(B)** Phosphorylation assay for NF-κB p65 on serine 536 in homozygous LCL at basal levels and after stimulation with TWEAK at 250 ng/mL. **(C)** Representative NF-κB western blot of homozygous LCL before and after treatment with 250 ng/mL of TWEAK. **(D)** NF-κB western blot showing significant differences between both genotype cells at basal levels as well as differences within the protective genotype cells before and after stimulation (*p < 0.05). CC, risk genotype; TT, protective genotype.

We measured total and phosphorylated NF-κB p65 on serine 536 in conditioned LCLs to determine if the variant rs4947296 had any effect on NF-κB phosphorylation. Non-stimulated LCLs with the risk genotype (CC) showed a significantly higher amount of total NF-κB when they were compared to cells with the protective genotype (TT) at basal levels (Figure 4A, *p* = 0.006). Thus, when we compared risk and protective genotypes in stimulated LCLs, we also found significant differences (*p* = 0.026). However, rs4947296 did not increase phosphorylation on S536 in NF-κB p65 subunit in the risk genotype, and the stimulation with TWEAK itself, did not increase NF-κB p65 phosphorylation in LCL (Figure 4B, for both comparisons, *p* > 0.05).

### The rs4947296 Upregulates the Translation of NF-κB in Lymphoblasts

Non-stimulated LCLs with the risk genotype (CC) showed a higher expression of *TNFRSF12A* and *NFKB1* RNA (3.6 ± 0.7 and 2.7 ± 0.7 fold higher, respectively) when they were compared to TT LCLs, confirming the previous results obtained in selected PBMC. When we stimulated both cell lines with 250 ng/mL of TWEAK, we found no significant differences for *TNFRSF12A*; however, the expression of *NFKB1* was significantly increased (CC: 10.4 ± 0.8; TT 3.7 ± 0.2, *p* = 1.4 × 10^−4^).

This finding was validated by western blot at protein level (Figures 4C,D) finding marginally significant differences when comparing risk and protective genotypes at basal levels (*p* = 0.05), but not after stimulation. When we compared each group before and after stimulation with 250 ng/mL of TWEAK, we found significant differences in the protective genotype (*p* = 0.017), but not in the risk genotype (*p* = 0.49), in accordance with the findings observed in ELISA.

**Figure 5:**
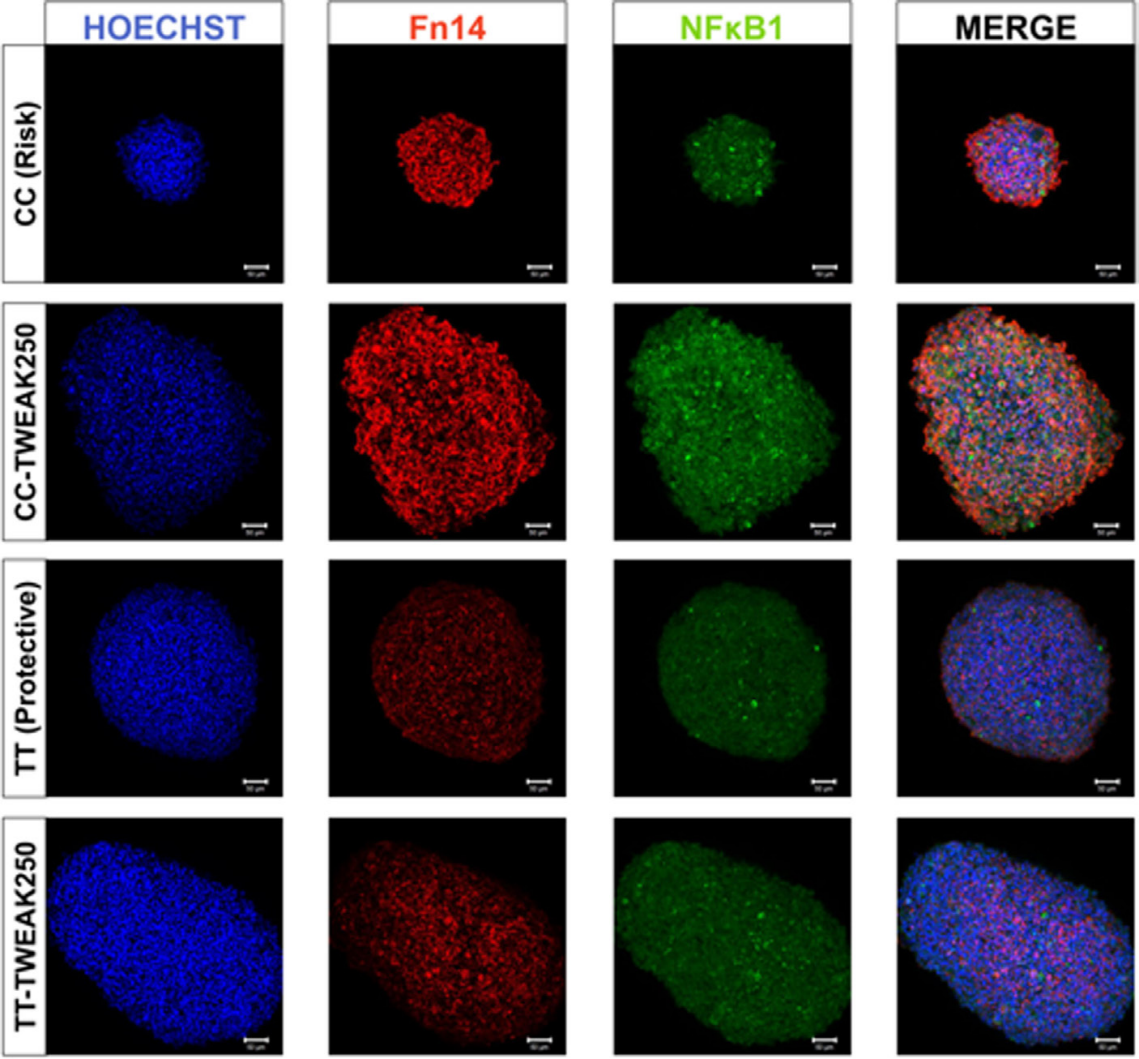
Fn14 and NF-κB expression in homozygous lymphoblastoid cell lines (LCLs). Confocal microscopy images showing representative clusters of LCLs with Fn14 and NF-κB immunolabeling after treatment with 250 ng/mL of TWEAK. CC, risk genotype; TT, protective genotype.

We also performed immunocytochemistry to quantify *TNFRSF12A* and *NFKB1* expression at protein level in LCLs by confocal microscopy (Figure 5). At basal levels, we found significant differences in the translation of Fn14 between both cell lines (CC: 78.5 ± 9.6; TT 48.3 ± 3.7, *p* = 10^−3^), but no differences were found for NF-κB (CC: 56.2 ± 4.2; TT 45.2 ± 3.3, *p* = 0.06). However, TWEAK upregulated the translation of NF-κB significantly in the risk LCLs (CC: *p* = 0.01; TT: *p* = 0.04), but it has no effect on Fn14 in neither of LCLs (CC: *p* = 0.77; TT: *p* = 0.29).

## Discussion

The main finding of this study is that the SNV rs4947296 is associated with bilateral MD. This variant is a trans-eQTL in lymphoid cells regulating gene expression in several genes in the TWEAK/Fn14 pathway and it activates NF-κB, probably increasing the inflammatory response in MD.

### Bilateral MD Is a Heterogeneous Disorder Including Five Clinical Variants

Bilateral MD is a severe, disabling inner ear condition, whose diagnosis usually requires few years of follow-up, since it is based on clinical criteria and no biological marker is available for its diagnosis (1). Moreover, BMD is a heterogeneous disorder that includes several clinical variants. A phenotype-driven cluster analysis has defined five subgroups of patients with potentially different etiology (39). BMD type 1 and type 2 are defined by diachronic or synchronic hearing loss, respectively, without migraine or AD; BMD type 3 includes familial MD cases and we have excluded them on this study; BMD type 4 is defined by migraine as a comorbid condition without AD, and BMD type 5 includes all patients with a comorbid AD. Since the prevalence of BMD is around 25% in our cohort and BMD type 5 is found in 11% of cases, we could estimate that the prevalence of BMD type 5 will be ≈1/40,000 individuals. Our results confirm previous studies that supported a significant association between BMD, migraine and ADs (12, 13). Here, we describe a locus at 6p21.33 suggesting association with BMD, being the leading signal rs4947296.

### The Variant rs4947296 Associated with BMD Is a Trans-eQTL and Regulates Several Genes in the TWEAK/Fn14 Pathway

Our results show that rs4947296 is an eQTL in mononuclear cells and it regulates the expression of 31/34 genes in the TWEAK/Fn14 signaling pathway (Table S2 in Supplementary Material) and 16/51 genes in the signal transduction NF-κB activation pathways (Table S3 in Supplementary Material). The SNV rs4947296 has been previously described as one the most strongly SNV associated with Behcet’s disease (*p* < 10^−12^) in a GWAS conducted in Korean, Japanese, and Han Chinese populations (40–42), as well as associated with Graves’ disease in Chinese population (43). Our study confirms that the rs4947296 is a trans-eQTL regulating gene expression in the Fn14/TWEAK pathway in lymphoid cells, and these findings support a role for an abnormal innate immune response in the pathophysiology of BMD. The TWEAK/Fn14 pathway has been involved in skin autoimmune disorders. So, TWEAK/Fn14 activation triggers Ro52-mediated photosensitization in cutaneous lupus erythematosus and involves the activation of NF-κB pathway (44). Furthermore, TWEAK/Fn14 contributes to the pathogenesis of bullous pemphigoid by reducing BP180 of hemidesmosomes and activating ERK and NF-κB pathways (45), demonstrating a pathogenic effect on the proteins of intercellular junctions.

In addition, TWEAK/Fn14 pathway could also be involved in Behcet and Graves’ disease and it could be a potential target for therapy is these disorders. So, pleiotropy is a common finding in trans-eQTL for autoimmune disorders (46, 47), and SNVs in the HLA region showing trans-eQTL effects were 10-fold enriched (48).

### The Variant rs4947296 Regulates NF-κB-Mediated Inflammation in Lymphoid Cells in BMD

TWEAK is a multifunctional cytokine that regulates multiple cellular responses, including angiogenesis, inflammation, cellular adhesion, proliferation, or apoptosis (49, 50). TWEAK activates signals through its receptor, Fn14, encoded by *TNFRSF12A* gene, which is highly expressed in epithelial cells and induced in several human diseases (51). High levels of TWEAK and/or Fn14 have also been found to be associated with the pathogenesis of rheumatoid arthritis (52), SLE (53), multiple sclerosis (54), or neuroinflammation (31). The binding of TWEAK to Fn14 induces both, an acute activation of the canonical NF-κB pathway and a prolonged activation of the non-canonical NF-κB pathway (49). Furthermore, the non-canonical NF-κB pathway plays a key role in immunity and immune-mediated disorders as SLE (49). Our findings using homozygous LCLs demonstrate that this eQTL upregulates the expression and translation of NF-κB in lymphoid cells and it may influence phosphorylation on S536 in the transactivation domain of NF-κB p65. Although our results were not statistically significant, they showed a trend for the risk genotype.

The non-canonical NF-κB pathway relies on the phosphorylation-induced p100 processing, which is triggered by signaling from a subset of TNFR members, including Fn14, TNFR2, BAFFR, CD40, LTβR, and RANK (55). Most of these signals are regulatory elements of the immune response and support the hypothesis that the allelic variants of genes of the immune response can modify the clinical course in MD. Previous studies have suggested that variants in *NFKB1* and *TLR10* genes are modifiers of hearing outcome in patients with uni (26) or BMD (56), but the relationship between TLR10 and NF-κB-mediated inflammation in MD is not known.

### The Site of NF-κB-Mediated Inflammation in MD Remains to be Defined: the Blood–Labyrinth Barrier (BLB), the Endolymphatic Sac, Fibrocytes of the Spiral Ligament, or/and the Tight Junctions (TJ) at the Reticular Lamina

This study provide evidences that the risk genotype could be used as predictor for bilateral SNHL in MD and our findings support an NF-κB-mediated inflammation in MD. In addition, this signal is a trans-eQTL and it regulates TWEAK/Fn14 pathway.

**Figure 6:**
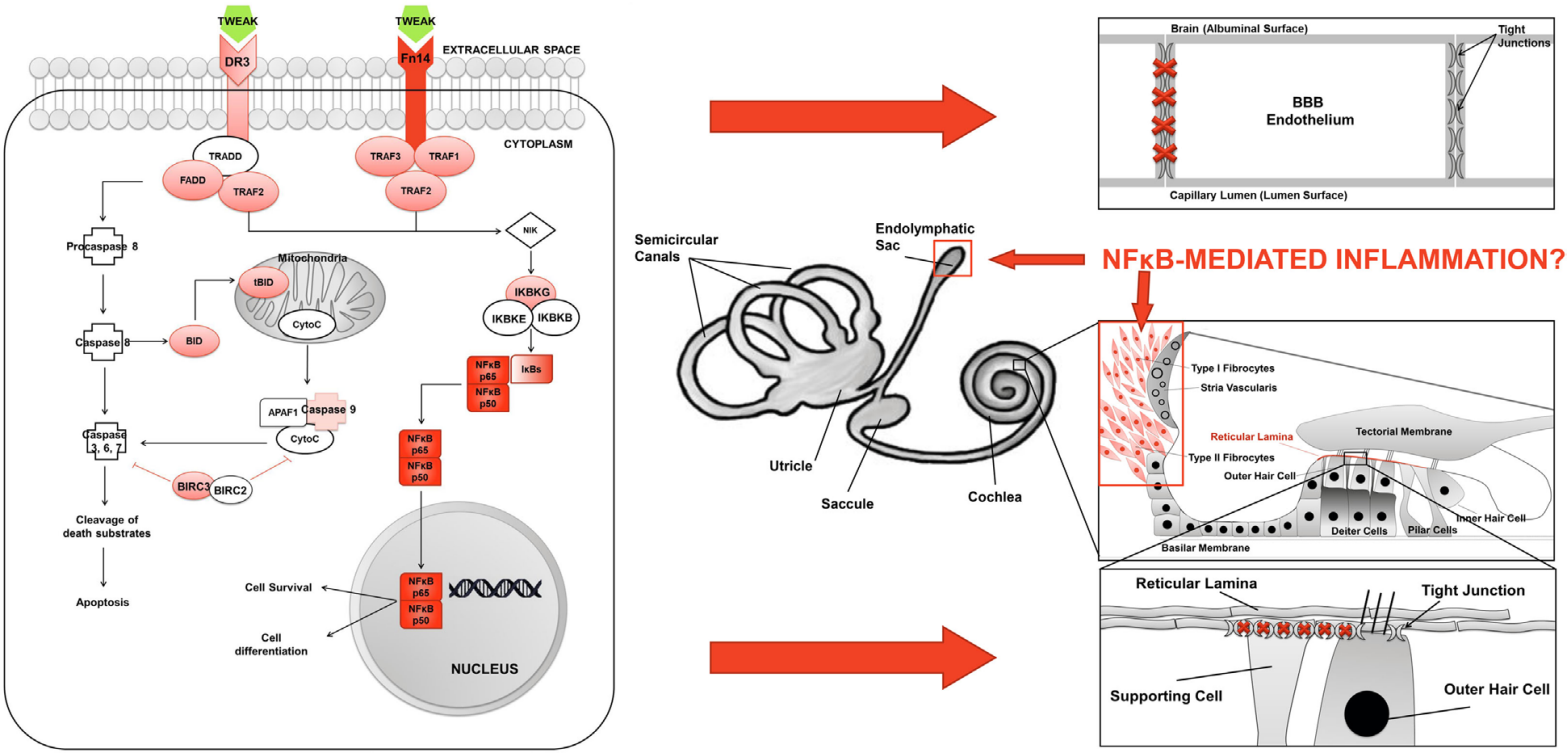
Inflammation model in Meniere’s disease (MD). **(A)** TWEAK/Fn14 pathway activates non-canonical NF-κB signaling in lymphoid cells in MD. **(B)** Potential sites of inflammatory damage are the blood–brain barrier (BBB), the endolymphatic sac, the spiral ligament, and the reticular lamina in the neurosensory epithelium of the cochlea.

Although TWEAK could induce the abnormal activation of this pathway in MD, the site of inflammation is unknown. An interesting hypothesis to explore is an inflammatory damage of the BLB, given the role of TWEAK in maintaining the blood–brain barrier (BBB) permeability and regulating the structure and function of the neurovascular unit (25) (Figure 6). Recent evidences suggest a role for TWEAK/Fn14 pathway in compromising the BBB in neuropsychiatric SLE (57). So, TWEAK/Fn14 interactions increase the accumulation of inflammatory cells in the choroid plexus, disorganizing BBB integrity and inducing neuronal death *in vitro* by the NF-κB signaling pathway (58, 59), but the role of TWEAK/Fn14 in the regulation of the BLB is unexplored.

A second hypothesis is that inflammation may occur in the endolymphatic sac, since proteomic studies have found a high content of immunoglobulins in the sac (15). The sac is a small organ located in the posterior cranial fossa and has a crucial role, not only in the maintenance of endolymph composition but also in the innate immune response (60). We hypothesize that, after exposure to an environmental trigger, the carriers of the risk genotype could have an abnormal NF-κB-mediated inflammatory response at the endolymphatic sac, causing an ionic imbalance in the endolymph leading to the accumulation of endolymph at the cochlear duct.

A third hypothesis will involve the increase of NF-κB in fibrocytes within the spiral ligament and the spiral limbus after a stress stimuli and the release of proinflammatory cytokines. Genetic mutations involving spiral ligament cells may lead to SNHL (61–63). Immune-mediated and acoustic trauma-mediated hearing impairment may result from the vulnerability of type I and type II fibrocytes to acoustic trauma and systemic inflammatory stress, respectively (64).

The last hypothesis affects cell adhesion molecules in the neurosensorial epithelium of the cochlea. The strict compartmentalization in the inner ear is necessary for normal hearing and is achieved by the TJs of the reticular lamina (65). An outstanding example for these interactions is established by the tight-junction proteins ZO-1, ZO-2, and ZO-3 that connect with the cytoplasmic domains of different integral membrane proteins such as occludins and claudins (66). TJP ZO-1 protein was shown to directly interact with F-actin, building a molecular bridge between integral membrane proteins like tricellulin (encoded by the *TRIC* gene) and the cytoskeleton, and human mutations in *TRIC* lead to deafness (67). Other members of the TJP have also been described to be involved in some types of deafness. Thus, a mutation in *TJP2* was linked to progressive NSHL DNFA51 (68). So, carriers of the risk genotype may have an abnormal expression of cell adhesion molecules, which may compromise the permeability of the reticular lamina causing an ionic imbalance.

## Conclusion

We present experimental data showing that the rs4947296 regulates gene expression in the TWEAK/Fn14 pathway in PBMCs and LCLs. This locus is a trans-eQTL and upregulates the translation of NF-κB in LCLs, supporting a regulatory effect in immune response.

Fn14 receptor and NF-κB are potential targets for drug therapy for carriers of the risk genotype in MD. Future preclinical studies and clinical trials using inhibitors of this pathway will be needed to demonstrate any potential benefit.

## Patent

JL-E. Use of allelic variants in the locus 6p21.33 for the diagnosis, prognosis and treatment of Meniere’s disease. Patent P201531458, October 9, 2015.

## Ethics Statement

The study was carried out in accordance with the recommendation of the Declaration of Helsinki of 1975 (as revised in 2013). The study protocol PI13/1242 with reference number 01-2014 was approved by the Ethical Review Board in Almeria and all the ethics committees for clinical research of all the recruiting centers. All participants gave written informed consent.

## Author Contributions

LF, TR, and AG-M performed experimental work including genotyping and gene expression arrays, cell culture, and confocal imaging studies. LF, TR, SO, AG-M, MM-B, AS, and JL-E performed statistical and bioinformatics analyses. IA, AB-C, JBDR, JE-S, JJFR, AG-A, RG-A, PM, EM-S, NP, PP, HP-G, SS-P, AS-V, MT, GT-R, and JL-E recruited patients and obtained informed consent in all individuals. MA-R and JL-E designed the study and data interpretation. JL-E supervised all experiments and LF and JL-E drafted the manuscript. All authors revised and approved the final version of the manuscript.

## Conflict of Interest Statement

The authors declare that the research was conducted in the absence of any commercial or financial relationships that could be construed as a potential conflict of interest.

## Footnotes

^1^https://genome.ucsc.edu/cgi-bin/hgLiftOver.

^2^http://www.ingenuity.com/products/ipa.

^3^https://portal.genego.com/.

^4^http://www.broadinstitute.org/mammals/haploreg/haploreg.php

^5^http://www.bios.unc.edu/research/genomic_software/seeQTL/.

^6^http://regulomedb.org/.
